# Magnetic resonance imaging of the knee in Norway 2002–2004 (national survey): rapid increase, older patients, large geographic differences

**DOI:** 10.1186/1472-6963-7-115

**Published:** 2007-07-22

**Authors:** Ansgar Espeland, Nils L Natvig, Ingard Løge, Lars Engebretsen, Jostein Ellingsen

**Affiliations:** 1 Department of Radiology, Haukeland University Hospital, N-5021 Bergen, Norway; 2 Section for Radiology, Department of Surgical Sciences, University of Bergen, Haukeland University Hospital, N-5021 Bergen, Norway; 3 Department of Radiology, Sykehuset Buskerud HF (Buskerud Hospital Trust), Dronninggt. 28, N-3004 Drammen, Norway; 4 Edda Legesenter, Eirik Jarls gt. 14, N-7030 Trondheim, Norway; 5 Department of Orthopaedics, Ullevål University Hospital, N-0407 Oslo, Norway; 6 Department of Statistics and Research, Directorate of Labour and Welfare, the Norwegian Labour and Welfare Organisation (NAV), Postboks 5, St. Olavs Plass, N-0130 Oslo, Norway

## Abstract

**Background:**

Magnetic resonance imaging (MRI) of the knee is the second most common MRI examination in Norway after head/brain MRI. Little has been published internationally on trends in the use of knee MRI after 1999. This study aimed to describe levels and trends in ambulant knee MRI utilisation in Norway 2002–2004 in relation to type of radiology service, geographic regions, number of MRI-scanners, patient age and gender, and type of referring health care provider.

**Methods:**

We analysed administrative data on all claims for reimbursement of ambulant knee MRI performed in Norway in 2002, 2003 and 2004 and noted nominal reimbursement. We also recorded the referring health care provider from clinical requests of ambulant knee MRI done consecutively during two months in 2004 at one private institute and three hospitals. Number of MRI-scanners was given by manufacturers and radiology services.

**Results:**

In Norway, the rate of knee MRI claims for 2004 was 15.6 per 1000 persons. This rate was 74% higher in East than in North region (18.4 vs. 10.6), slightly higher for men than women (16.4 vs. 14.7) and highest for ages 50–59 years (29.0) and 60–69 years (21.2). Most claims (76% for 2004) came from private radiology services. In 2004, the referring health care provider was a general practitioner in 63% of claims (unspecified in 24%) and in 83.5% (394/472) of clinical requests. From 2002 to 2004, the rate of knee MRI claims increased 64%. In the age group 50 years or above the increase was 86%. Rate of MRI-scanners increased 43% to 21 scanners per million persons in 2004. Reimbursement for knee MRI claims (nominal value) increased 80% to 70 million Norwegian kroner in 2004.

**Conclusion:**

Ambulant knee MRI utilisation in Norway increases rapidly especially for patients over 50, and shows large geographic differences. Evaluation of clinical outcomes of this activity is needed together with clinical guidelines for use of knee MRI.

## Background

Published data indicate a large increase during the nineties in the use of magnetic resonance imaging (MRI) [1], including extremity [2] and specifically knee MRI [3]. The criteria for performing MRI of the knee have broadened considerably [3]. Possible advantages are improved detection of relevant traumatic lesions [4] and reduced use of invasive diagnostic arthroscopy [2,5], as MRI provides good visualisation of menisci and ligaments [6,7].

The overall clinical benefit of the current use of knee MRI is uncertain, however [8-10], and overuse may exist. Irrelevant findings such as degenerative rupture of the medial meniscus are frequent, especially in middle and older ages [11-13]. A study [14] from Wales published 2002 found that 46% of knee MRI requests were not regarded as clinically indicated.

According to The Norwegian Radiation Protection Authority (NRPA), knee MRI, with 10.7 examinations per 1000 persons, was the second most frequent MRI examination in Norway in 2002 after head/brain MRI [15]. Knee MRI examinations varied more in frequency between geographical regions than did any other of the 30 most common radiological examinations. Further published international or Norwegian data on knee MRI utilisation after 1999 are scarce.

In Norway, nation-wide studies of radiology utilisation are feasible, as data are stored on all claims for reimbursement of ambulant radiology services. We have analysed data for examinations performed 2002–2004. Our aim was to describe levels and trends in ambulant knee MRI utilisation in Norway 2002–2004 in relation to type of radiology service, geographic regions, number of MRI-scanners, patient age and gender, and type of referring health care provider.

## Methods

This was a survey of all claims for reimbursement of ambulant knee MRI done in Norway 2002–2004. We also reviewed clinical requests for ambulant knee MRI performed 2004.

### Setting

In Norway, general practitioners (GPs), other physicians, chiropractors, and from 2006 also manual physiotherapists all have direct access to both public and private knee MRI services. GPs have a "gatekeeper" role towards secondary care specialists such as orthopaedic surgeons.

NAV – the Norwegian Labour and Welfare Organisation (earlier RTV) – reimburses public and private ambulant (but not in-patient) radiology services and files all reimbursement claims in a quality-assured database. From 2002 such claims contain codes for modality (e.g. MRI) and localisation (e.g. knee) and may also contain other codes from the Norwegian radiology coding system NORAKO [16]. One claim concerns one consultation with one or more examinations (e.g. knee MRI, knee radiography), each with one or more codes. Reimbursement is based on grouping of codes, associated cost weights and a fixed "average" examination price or unity price regularly set by the Ministry of Health and Care Services.

Payment for ambulant radiology services in Norway is not restricted to reimbursement (reported in this study). Public funding further provides a basic support for public services and a partial fee-for-service for private services [17]. The patient pays a fixed price per ambulant consultation (2004: 185 or 215 Norwegian kroner (NKr)), but zero after his/her total health care expenses the actual year exceeds a fixed upper limit (2004: 1550 NKr).

### Data

NAV provided data on all claims for reimbursement of knee MRI (code MR GE) done in 2002, 2003 and 2004. Variables included number of claims, reimbursement, radiology service (public, private), geographic health region based on patient address (North, Central, West, South, East), patient age and gender, and type of referring health care provider.

Referring health care provider was also noted directly from clinical requests for ambulant knee MRI performed consecutively in October/November 2004 at one private institute and three hospitals of different size. Number of MRI-scanners in each region each study year was obtained from manufacturers and radiology services.

### Analysis

Annual rate of knee MRI claims was computed as number of claims per 1000 and million persons based on population data from Statistics Norway [18]. Annual rate of MRI-scanners was registered as mean number of scanners each year per million persons. Since many scanners were located at the border between regions South and East (in Oslo, capital of Norway), these two regions are grouped together when presenting number of scanners.

Reimbursement is presented in NKr nominal value as reported by NAV. Mean conversion rates in 2002, 2003 and 2004 were 7.9702 NKr, 7.0824 NKr and 6.7372 NKr for 1 US Dollar and 7.5073 NKr, 8.0039 NKr and 8.3715 NKr for 1 Euro, respectively.

Multivariate linear regression analysis was used to assess a potential relation between rate of claims (number of claims per million persons – dependent variable) and scanner rate, after adjusting for year and geographic region. Univariate linear regression analysis was applied to evaluate a possible association between rate of claims and the ratio between the rate of claims for the age group of 50 years or above and for the age group below 50 years. Mantel-Haenszel chi-square test was used to compare rates between years. Breslow-Day's test of homogeneity of odds ratios (ORs) was applied to compare ORs between years. Excel and SPSS were used to analyse data. *P *< 0.05 was regarded to indicate statistical significance.

No person-identifiable data were analysed or recorded in this study. According to The Regional Committee for Research Ethics in Western Norway, the study did not require approval from a research ethics committee.

## Results

### Ambulant knee MRI in Norway 2002–2004

The number of claims for reimbursement of ambulant knee MRI done in Norway in 2004 was 71240 (Table 1) – or 15.6 claims per 1000 persons, which was a 64% increase from 2002 (Table 2). The reimbursement increased 80% to 70 million NKr in 2004 (Table 1).

**Table 1:** Claims and reimbursement for ambulant knee MRI in Norway, by type of radiology service

	2002 n (%)	2003 n (%)	2004 n (%)	Change in n 2002–2004, %
Claims				
Private	32 708 (76)	43 782 (73)	54 039 (76)	+ 65
Public	10 132 (24)	16 283 (27)	17 201 (24)	+ 70
Total	42 840 (100)	60 065 (100)	71 240 (100)	+ 66
Reimbursement*				
Private	30 177 (77)	34 132 (73)	55 942 (80)	+ 85
Public	8 870 (23)	12 524 (27)	14 358 (20)	+ 62
Total	39 047 (100)	46 656 (100)	70 300 (100)	+ 80

MRI = magnetic resonance imaging.

* In thousand Norwegian kroner, nominal value.

**Table 2:** Claims for ambulant knee MRI in Norway, by geographic region

	n (rate = n per 1000 persons in region)			Change in rate* from 2002 to 2004, %
		
	2002	2003	2004	
North	3 339 (7.2)	3 907 (8.4)	4 895 (10.6)	+ 47
Central	1 884 (3.0)	5 635 (8.8)	8 715 (13.6)	+ 359
West	3 599 (3.9)	7 877 (8.4)	12 079 (12.8)	+ 231
South	11 346 (12.9)	14 664 (16.5)	15 425 (17.3)	+ 35
East	22 672 (14.0)	27 987 (17.2)	30 126 (18.4)	+ 31
Norway	42 840 (9.5)	60 065 (13.2)	71 240 (15.6)	+ 64

MRI = magnetic resonance imaging.

For 2002, 2003 and 2004, respectively, 263, 202 and 235 claims with unspecified region have been included under specified regions, according to the relative number of claims for patients from these regions before inclusion.

* Mantel-Haenszel chi-square test for linear trend in change: p < 0.001 for all regions.

### Comparison between private and public radiology services

Private radiology services contributed about three-quarters of the reimbursement claims each year during the study period. In 2004, they received 80% of the reimbursement (Table 1).

### Relation to geographic regions and number of MRI-scanners

In 2002, the rate of knee MRI claims differed more than fourfold between regions and was highest in East (Table 2). The difference declined from 2002 to 2004. By 2004 the rate was nevertheless 74% higher in East than in the North region (18.4 vs. 10.6, Table 2).

The mean number of MRI-scanners in Norway during 2002, 2003 and 2004 was 66.5, 83.5 and 96.0, respectively. The rate of MRI-scanners (mean number per million persons) was 43% higher in 2004 than in 2002 (21.0 vs. 14.7, Table 3). In 2004, this rate was 80% and 67% higher in Central and South/East regions, respectively, compared to the West region (24.9 and 23.1 vs. 13.8, Table 3).

**Table 3:** MRI-scanners in Norway, by geographic region

	Mean* (rate = mean per million persons in region)			Change in rate# from 2002 to 2004, %
		
	2002	2003	2004	
North	7.5 (16.2)	8.0 (17.3)	8.5 (18.4)	+ 13
Central	8.5 (13.3)	13.5 (21.1)	16.0 (24.9)	+ 87
West	7.0 (7.6)	9.5 (10.2)	13.0 (13.8)	+ 83
South/East	43.5 (17.4)	52.5 (20.9)	58.5 (23.1)	+ 33
Norway	66.5 (14.7)	83.5 (18.3)	96.0 (21.0)	+ 43

MRI = magnetic resonance imaging.

* Mean number of scanners each year is mean of numbers 1. January same and next year.

# Mantel-Haenszel chi-square test for linear trend in change: p = 0.03 for Norway, p = 0.81 for region North and p = 0.16–0.19 for the other regions.

Rate of knee MRI claims was significantly related to rate of MRI-scanners when adjusting for year and geographic region in a multivariate linear regression analysis (p = 0.017, Table 4). Regions with fewer scanners (West, North) (Table 3) had generally lower knee MRI utilisation (Table 2).

**Table 4:** Rate of knee MRI claims by rate of MRI-scanners, adjusted for year and geographic region: linear regression analysis

Explanatory variable	Regression coefficient	95 % CI
Rate of MRI-scanners*	744	201, 1287
Year		
2002	-2046	-6024, 1931
2003	-1081	-3490, 1328
2004 (reference)	0	
Geographic region		
North	-5079	-7887, -2271
Central	-7234	-9478, -4991
West	-400	-6242, 5443
South/East (reference)	0	

MRI = magnetic resonance imaging.

CI = confidence interval.

* P = 0.017. Intercept (95% CI) = 2003 (-10906, 14913).

### Utilisation by patient age and gender

Claims for knee MRI were most frequent and also most rapidly increasing in the age group 50–59 years (Table 5). The rate of knee MRI claims differed only slightly between genders (Table 5).

**Table 5:** Claims for ambulant knee MRI in Norway, by patient age and gender

	n (rate = n per 1000 persons in age or gender group)			Change in rate* from 2002 to 2004, %
		
	2002	2003	2004	
Age, years				
<20	4 409 (3.8)	6 528 (5.5)	7 753 (6.5)	+ 74
20–29	6 185 (10.5)	8 052 (13.9)	8 630 (15.1)	+ 44
30–39	8 139 (11.8)	11 449 (16.4)	12 180 (17.4)	+ 48
40–49	8 518 (13.5)	11 312 (17.8)	12 857 (20.1)	+ 49
50–59	8 491 (14.8)	12 358 (21.1)	17 229 (29.0)	+ 95
60–69	4 337 (12.2)	6 446 (17.8)	7 964 (21.2)	+ 74
>70	2 761 (5.4)	3 920 (7.7)	4 627 (9.1)	+ 69
Gender				
Men	23 269 (10.4)	32 101 (14.2)	37 310 (16.4)	+ 58
Women	19 571 (8.6)	27 964 (12.2)	33 930 (14.7)	+ 71

MRI = magnetic resonance imaging.

For 2002, 2003 and 2004, respectively, 1, 7 and 231 claims with unspecified age and 1791, 1187 and 614 claims with unspecified gender have been included under specific age and gender groups according to the relative number of claims in these groups before inclusion.

* Mantel-Haenszel chi-square test for linear trend in change: p < 0.001 for all groups.

Compared to the rate of claims for ages below 50, the rate of claims for older ages was 23% higher in 2002 (OR 1.23), 30% higher in 2003 (OR 1.30) and 52% higher in 2004 (OR 1.52) and thus also increased more (p < 0.001). The increase from 2002 to 2004 was 86%. In 2004, regions with a higher total rate of claims generally had a higher ratio between the rate of claims for ages above 50 and the rate of claims for ages below 50 (East 1.56, South 1.59, Central 1.52, West 1.45, North 1.07) (p < 0.001, univariate linear regression).

### Type of referring health care provider

The referring health care provider was unspecified in 77% of claims for 2002 and in 46% of claims for 2003. It was a GP in 63% of claims for 2004 but unspecified in 24%. Review of clinical requests of 472 ambulant knee MRI examinations done at four institutions during two months in 2004 showed that GPs had ordered 394 (83.5%), orthopaedic surgeons 58 (12.3%) and other specified groups 20 (4.2%) examinations.

## Discussion

This population-based study showed a very rapid increase in knee MRI utilisation. Claims for ambulant knee MRI increased 64% in two years to 15.6 per 1000 persons in 2004. Utilisation of knee MRI was higher for patients above 50, differed considerably between regions, was related to number of MRI-scanners, and was most often initiated by a GP.

### Strengths and limitations

The coding of modality (MR) and location (GE) identifying knee MRI claims in this nation-wide study is likely to be quite complete. Radiology services had used such coding for many years to monitor activity, before it became required for reimbursement from 2002. NRPA has collected lists of radiology use for the whole year 2002 from all radiology services in Norway; 99.2% of all listed examinations were coded for both modality and location [15].

The present data on geographic region, age and gender are also nearly complete for each study year. Geographic region, age and gender was specified in at least 99.4%, 99.7% and 95.8% of claims, respectively (based on numbers in footnotes of Tables 2 and 5).

Our data on number of claims are not affected by number of radiological codes. NRPA estimated number of MRI examinations from number of codes, excluding codes for MRI sequences but not other procedure codes amounting to 9% of MRI codes [15]. This probably implied slight overestimation of the number of MRI examinations. Our results on geographic differences, as opposed to most of the NRPA data, are based on patient address and thus reflect who are examined rather than where the examination took place. Also, different from NRPA, we report on reimbursement, scanners, patients, and referring health care providers.

Unfortunately, many claims did not specify the profession of the referring health care provider. We therefore reviewed clinical requests for this purpose. The study gave no information on clinical diagnoses, MRI findings or clinical outcomes.

### Interpretation of findings

Medical needs for knee MRI are unlikely to differ as much as 74% between regions in Norway, as did the rate of knee MRI claims. Thus, regional under- and/or overuse of knee MRI might exist. In total, overuse seems likely. We found extensive use in older age groups where osteoarthritis is the dominating knee disorder. Knee MRI is not recommended in most cases of suspected osteoarthritis. It is mainly indicated for selected cases of acute trauma, especially in younger patients, and in selected patients prior to arthroscopy [11,19-22].

### Potential explanations

Several explanations for the rapid increase in use of knee MRI can be considered. First, the indications for knee MRI must have broadened, since neither the increase nor the larger increase for ages above 50 can be fully explained by increased disease prevalence.

Second, more MRI-scanners in the patient's health region were associated with more frequent use of knee MRI. Distance to the radiology clinic and illness severity explained 39% of the variation in use of MRI and computed tomography in a study from USA [23].

Third, use of knee MRI increased relatively more than number of MRI-scanners. This means increased knee MRI activity per scanner, on average. Dedicated extremity low-field scanners are now common and constituted 16 of 103 MRI-scanners in Norway per 1. January 2005.

Fourth, many patients ask for or demand imaging, and physicians may find it difficult or unwise to resist [24,25]. More and more patients want to take part in clinical decisions [26]. At the same time, GPs in Norway are becoming less willing to be "gatekeepers" [27,28] and may thus be more likely than before to meet patients' wishes for diagnostic tests.

Fifth, several other contextual factors may also affect the use of imaging tests [24]. E.g., GPs may order knee MRI to facilitate, or prevent, referral to an orthopaedic surgeon [29].

### Comparison with other studies

The larger increase in knee MRI utilisation in our study for patients above 50 conflicts with findings from USA where the age of patients undergoing knee MRI did not change from 1991 to 1995 [3]. Shift in utilisation towards older age groups may be a recent phenomenon.

Our finding of 9.5 ambulant knee MRI claims per 1000 persons in 2002 compares well with the NRPA estimate of 10.7 ambulant and non-ambulant knee MRI examinations [15]. Knee MRI in Norway in 2002 was thus about 60% more frequent than lower extremity MRI in the USA Medicare population in 1999 (10.7 vs. 6.6 examinations per 1000 persons) [2,15]. Further comparable data after 1999 or outside USA are not available to our knowledge.

### Implications

We suggest four implications of our findings. First, the use of knee MRI should be further monitored. In Norway, NAV's administrative data are suitable for this. Second, current reasons and indications for ordering knee MRI should be identified. This may require review of MRI requests and interviews with clinicians and patients. Third, the present use of knee MRI should be evaluated against evidence-based guidelines or other appropriateness criteria [30]. Fourth, as basis for up-dated guidelines, more evidence is needed on the effects of today's use of knee MRI on treatment, outcomes and costs [31].

## Conclusion

Ambulant knee MRI utilisation in Norway increases rapidly, especially among patients aged 50 years or above, and it differs considerably between geographic regions. The indications, clinical outcomes and appropriateness of this activity should be evaluated. Up-dated clinical guidelines for use of knee MRI should be developed and implemented.

## Competing interests

The author(s) declare that they have no competing interests.

## Authors' contributions

AE participated in the conception and design of the study, collected data, performed statistical analyses, and drafted the manuscript. NLN participated in the conception and design of the study, collected and interpreted data, and helped to revise the manuscript. IL and LE both participated in the conception and design of the study, interpreted data, and helped to revise the manuscript. JE extracted data from the NAV database, interpreted data, and helped to draft the manuscript. All authors read and approved the final manuscript.

## Pre-publication history

The pre-publication history for this paper can be accessed here:


